# Acute Pancreatitis as a Trigger for Thrombotic Microangiopathy: A Case Report

**DOI:** 10.7759/cureus.20103

**Published:** 2021-12-02

**Authors:** Francisco Adragão, Inês Nabais, Rúben Reis, Bernardo Pereira, Armindo Ramos

**Affiliations:** 1 Internal Medicine, Centro Hospitalar Universitário Do Algarve - Unidade Hospitalar de Portimão, Portimão, PRT; 2 Internal Medicine, Hospital de Cascais Dr. José de Almeida, Cascais, PRT; 3 Internal Medicine, Centro Hospitalar Barreiro Montijo, Barreiro, PRT; 4 Critical Care Medicine, Hospital de Cascais Dr. José de Almeida, Cascais, PRT

**Keywords:** acute pancreatitis, thrombotic microangiopathy, hemolytic uremic syndrome, multiple organ dysfunction syndrome, plasmapheresis

## Abstract

Thrombotic microangiopathies (TMA) are a group of disorders characterized by generalized microvascular occlusion, thrombocytopenia, and microangiopathic hemolytic anemia, which may present with organ dysfunction. These include hemolytic uremic syndrome (HUS) and thrombotic thrombocytopenic purpura (TTP) among others. The triad of anemia, thrombocytopenia, and acute kidney injury is the hallmark of HUS. It can be associated with Shiga toxin-producing *Escherichia coli* infection, complement-mediated (atypical HUS), coagulation or metabolism-mediated (predominantly in children of less than one year of age), or secondary HUS with the coexisting disease. HUS is a potentially fatal condition irrespective of its cause, and hence the diagnosis and management approach must be swift. The treatment is support-based; however, in severe cases, the use of plasmapheresis has shown favorable outcomes.

In this report, we discuss a case of a 30-year-old male who presented with acalculous acute pancreatitis with HUS, a rare case of secondary HUS previously reported in a few case reports.

## Introduction

Hemolytic uremic syndrome (HUS) is part of a group of disorders characterized by microangiopathic hemolytic anemia, thrombocytopenia, and acute kidney injury as a clinical hallmark. Although more common in children, it can also affect adults and may lead to a grimmer outcome. Acute pancreatitis is a rare, but possible trigger of HUS.

## Case presentation

A 30-year-old male with a medical history of hypertension, chronic kidney disease, unipolar major depression, and alcoholism, with no known prescribed medication, presented to the Emergency Department with an early-onset, dull, upper abdominal pain irradiating to the back, with gradual intensity. Accompanying symptoms included anorexia and acute diarrhea that had started the days before the presentation. No blood loss, mucus, nausea or vomiting, fever, or any other symptoms during this period were reported.

At admission, he was dehydrated, with a tender abdomen, painful palpation of the epigastrium but without peritoneal reaction. Vital signs registered were as follows: noninvasive blood pressure of 149/82 mmHg, heart rate of 110 beats per minute, respiratory rate of 16 cycles per minute, oxygen peripheral saturation of 94%, tympanic temperature of 37.1 ºC. Initial laboratory results returned were as follows: hemoglobin of 14.2 g/dL, white blood cells count of 11.4 x 10^9^ cells/L, 290 x 10^9^ platelets/L, serum amylase of 140 U/L, urea of 33 mg/dL, and creatinine of 1.52 mg/dL. Abdominal ultrasonography revealed enlarged liver, mild steatosis, no vesicular lithiasis, and pancreas without any gross changes. Abdominal CT scan showed diffuse densification of adipose tissue as well as peripancreatic fluid. Kidney size was asymmetric due to left kidney atrophy. Given the clinical presentation, past medical history, and ongoing evolution, the patient was admitted to the infirmary with the admission diagnosis of acute acalculous pancreatitis in relation to alcohol consumption.

Less than 12 hours after the admission, and despite adequate management, the patient developed progressive organ dysfunction. At the time of referral to the ICU, the patient was described as tachycardiac, oliguric, and with a depressed state of consciousness with a spontaneous eye response, confused speech, and localized pain (Glasgow Coma Scale score of 13). Laboratory findings at this time included hypochromic microcytic anemia, thrombocytopenia with 28 x 10^9^ platelets/L, a slight rise of total bilirubin of 1.14 mg/dL and direct bilirubin of 0.43 mg/dL, lactate dehydrogenase (LDH) of 1,307 U/L, and marked worsening of creatinine with a sudden rise to 7.61 mg/dL. A cranial CT scan revealed no relevant changes.

The patient was admitted to the ICU with an initial diagnosis of acute pancreatitis with multiple organ dysfunction syndrome (MODS), with predominantly neurological, hematological, and renal dysfunctions, and with established acute kidney injury [Kidney Disease Improving Global Outcomes (KDIGO) III]. During the ICU stay, the patient remained under oxygen therapy within [94-98%] targets to a maximum oxygen delivery of 0.35 FiO_2_; he was hemodynamically stable for the most part. Renal replacement therapy with intermittent hemodialysis was initiated on the first day of admission for acidemia and uremia control, with partial kidney function recovery by the third day of ICU stay.

The timing of onset of MODS and the persistence of renal and hematological dysfunctions, despite his inflammatory improvement, in the absence of cardiovascular attainment, led to further investigations. An extensive study of anemia and thrombocytopenia was performed, highlighting a blood smear with the presence of schizocytes and erythrocytes as target cells as well as platelet anisocytosis, normal blood clotting tests, d-dimers of 3 mg/L, and fibrinogen of 458 mg/dL. Further laboratory testing returned total bilirubin of 1.77 mg/dL, LDH of 1,378 U/L, normal haptoglobin, negative Coombs test, normal complement levels, negative Shiga toxin test, and ADAMTS13 with 53% activity.

Due to suspicion for HUS, the patient underwent plasmapheresis (PEX) for five consecutive days, with the progressive recovery of previous dysfunctions, eventually achieving a sustained increase in platelet levels. The improvement in renal dysfunction was only partial, maintaining the need for dialysis without any demand for other organ support; hence, the patient was transferred to the Nephrology Department, where he remained hospitalized for a few more days, remaining on dialysis protocol. After stabilizing his kidney function, he was discharged and referred to a chronic kidney disease consultation for follow-up.

## Discussion

HUS was first described in 1955. It is a thrombotic microangiopathy (TMA) characterized by generalized microvascular occlusion by platelet thrombi, thrombocytopenia, and microangiopathic hemolytic anemia. In the presence of renal and/or neurological dysfunctions/impairment, two main differential diagnoses that are often indistinguishable should be considered: HUS and thrombotic thrombocytopenic purpura (TTP) [1]. Among these, the HUS associated with Shiga toxin-producing *Escherichia coli* infection (STEC-HUS) is the most frequent, followed by TTP, atypical HUS (aHUS), and finally secondary HUS [1,2].

In the past years, a new classification for TMA was proposed, distinguishing between primary and secondary causes [3,4]. TMA caused by primary causes results from hereditary or acquired deficits, such as deficiency of ADAMST13 or complement gene abnormalities, while secondary TMA occurs due to other underlying conditions. Notwithstanding its etiology, TMA, if missed to diagnose and left untreated, results in high morbidity and mortality [5].

The triad of anemia, thrombocytopenia, and acute kidney injury is the hallmark of HUS. As in other TMAs, the damage occurs with a normal prothrombin time and activated normal partial thromboplastin time and nonimmune thrombocytopenia [5]. During the diagnostic workup, once thrombocytopenia and microangiopathic hemolytic anemia are confirmed, it is essential to rule out the presence of Shiga toxin as well as to measure ADAMTS13 activity. The differential diagnosis should hence take into account the following factors: the decrease in ADAMTS13 activity to values below 10% points in TTP; the presence of Shiga toxin in STEC-HUS; and, in the absence of toxins and a ≥10% ADAMTS13 activity, aHUS (in which there is a complement dysregulation) or HUS secondary to another disease (as in the case described above) should both be considered [4].

Pancreatitis as a cause for HUS has been previously reported, although it seems to be a rare etiology, with very few cases reported in the literature so far. In a systematic review of 21 cases published in 2007, each describing a patient who developed TMAs after acute pancreatitis, the majority of cases were men, with most of them related to alcohol consumption or gallbladder disease. In all of these cases, there was no evidence of TMAs at the time when pancreatitis was diagnosed, with a median duration of three days until the diagnose of TMAs was finally made. ADAMTS13 activity has only been reported in some cases, most of them with a ≥10% activity. More than half of them resolved after the diagnosis was made [6]. The mechanism that leads to a TMA is still not fully understood [7]. Multiple mechanisms have been proposed, and one among them states that pancreatic dysfunction and the inflammatory condition associated with it lead to changes in the von Willebrand factor and ADAMTS13, allowing spontaneous binding of platelets, and thereby promoting platelet aggregation [6,7].

Regardless of its etiology, it is important to bear in mind that HUS is a potentially fatal condition whose clinical presentation might belie an underlying organ dysfunction related to another pro-inflammatory condition, as was the case with our patient. The treatment of HUS is mainly focused on support. However, as stated in previously published reports, the use of PEX is highly effective in severe cases [6,7].

## Conclusions

In conclusion, as described in this case report and in line with previous findings in the literature, the majority of patients who developed TMA due to acute pancreatitis presented with clinically overlapping conditions, characterized by rapidly progressing MODS, and with subsequent need for early ICU admission. It is therefore important for doctors to recognize TMA as one of the potential causes of acute renal failure among adult patients with acute pancreatitis, especially in the setting of anemia and thrombocytopenia.
